# Continuous-flow processes for the catalytic partial hydrogenation reaction of alkynes

**DOI:** 10.3762/bjoc.13.73

**Published:** 2017-04-20

**Authors:** Carmen Moreno-Marrodan, Francesca Liguori, Pierluigi Barbaro

**Affiliations:** 1 Consiglio Nazionale delle Ricerche, Istituto di Chimica dei Composti Organo Metallici, Via Madonna del Piano 10, 50019 Sesto Fiorentino, Firenze, Italy

**Keywords:** alkynes, heterogeneous catalysis: hydrogenation, flow, liquid-phase

## Abstract

The catalytic partial hydrogenation of substituted alkynes to alkenes is a process of high importance in the manufacture of several market chemicals. The present paper shortly reviews the heterogeneous catalytic systems engineered for this reaction under continuous flow and in the liquid phase. The main contributions appeared in the literature from 1997 up to August 2016 are discussed in terms of reactor design. A comparison with batch and industrial processes is provided whenever possible.

## Introduction

The catalytic partial hydrogenation of alkynes to alkenes in the liquid phase is a reaction of high relevance to the manufacture of a multitude of fine chemicals [1] including pharmaceutical building blocks, agrochemicals, food additives, flavours and fragrances [2–3]. It is also crucial in the bulk polymer industry to achieve the complete elimination of alkynes and alkadienes from alkene feedstocks [4–5]. The chemistry of these processes is dominated by heterogeneous palladium catalysts, particularly based on solid-supported Pd nanoparticles (PdNP) [6–7]. On the industrial scale, alkynes partial hydrogenations are usually carried out under batch conditions using Lindlar-type catalysts, consisting in relatively high amounts of Pd (5 wt %) and Pb (2–3%) deposited onto CaCO_3_ [8–9], whose active sites nature is not fully characterized yet [10–11]. Besides the use of toxic lead, satisfactory catalyst performances often require a careful control of the hydrogen uptake and use of an excess of amine (quinoline) modifier [12–13], with serious drawbacks in terms of process economy, environmental impact and product separation management. The development of cost-effective, well-defined, efficient and environmentally friendly catalytic systems for the partial hydrogenation reaction of alkynes is thus of utmost importance [14–15].

Compared to batch setups, considerable process intensification [16–17] to this regard can be provided by continuous-flow operations either in terms of safety, purification, waste emission, durability, reproducibility, automation, energy and space consumption [18–19]. Particularly, continuous-flow catalysis may enhance the performance of a given catalyst while reducing the number of processing steps [20–21], which may result in a significant contribution to the reduction of the high E-factor (kg waste generated/kg product) usually observed in the fine-chemicals sector [22–23], as a consequence of the additives and manipulations required to achieve satisfactory selectivity. Indeed, the implementation of continuous-flow practices in the pharmaceutical industry is considered one of the most strategic fields of innovation toward greener manufacturing methods [24–25]. Nonetheless, in order to be competitive on the large-scale, continuous-flow systems for the catalytic hydrogenation of alkynes should not only provide their intrinsic benefits over conventional batch processes, but also be advantageous, or at least equal, either in terms of productivity per unit active metal, volume or time, absence of additives or catalyst lifetime [26–27].

In the present paper we shortly review the heterogeneous catalytic systems engineered for the partial hydrogenation reaction of substituted and unsubstituted alkynes under continuous flow and in the liquid phase, covering the main contributions appeared in the literature from 1997 up to August 2016. Some aspects of the topic have been surveyed in the past [28–29]. Details of alkyne hydrogenation reactions in general, including mechanism [30–31], kinetics [32–33], adsorption phenomena [34–35], thermodynamics [36–37], structure–activity relationships [38–39] have been extensively described elsewhere, therefore they are out of the scope of this review. Herein the focus is on the various catalytic reactor systems and technological solutions reported in the literature for this process, aimed at illustrating the state of the art in the field and the benefits of the approach. Generalities on theory and methods for flow chemistry can be found in excellent textbooks and reviews [40–41] and will not be treated in detail.

The present review could have been structured according to different variables, i.e., the metal catalyst involved, the type of support material, the reactor design or the hydrogen source. We decided to break down the manuscript on the basis of the substrates examined in order to allow an easier comparison among different reactors performance and highlight the potential benefits of one catalytic system over the others.

## Review

### The issue of selectivity

**Scheme 1:**
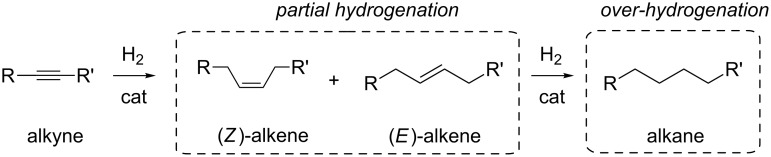
Common reaction pathways for alkyne hydrogenation reactions.

A major challenge in alkynes partial hydrogenation is to achieve 100% selectivity to the desired product at the highest conversion level [42–43]. The conventional heterogeneous catalysts often show selectivity issues owing to many potential side reactions, particularly in relation to chemoselectivity, i.e., over-hydrogenation of alkenes to alkanes [44–45], resistance of other functional groups (ketones [46–47], amines [48–49], azides [50]), regioselectivity [51–52] , isomerization [53–54] and oligomerization [55–56] competitive reactions. In addition, whenever an internal alkyne is to be hydrogenated, stereoselectivity must also be considered [57] (Scheme 1). The main byproduct usually obtained is the over-hydrogenation one, which results in conversion and selectivity to be inversely proportional. Selectivity in partial hydrogenation is ruled by the relative rates of the first and second hydrogenation steps, as well as by the adsorption strengths of alkyne and alkene over the metal catalyst surface. Other side-products may include those due to dimerization and isomerization reactions, depending on the substrate.

Several approaches were developed to enhance the selectivity of the batch hydrogenation processes, including tuning of the reaction conditions, use of less conventional metals [58–59], alloys [60–61] or oxide active phases [62] and engineering of single-site heterogeneous catalysts [63–64]. A more usual strategy is the so-called “selective poisoning”, i.e., the improvement of the catalyst’s selectivity by the addition of variable, often large, amount of contaminants, either organic ligands (quinolone [65–66], phosphine [67]), carbon monoxide [68], sulfides [69], sulfoxides [70–71] defined as “reaction modifiers”, or metal ions (Cu, Pb) [72], polymers/surfactants [73] defined “catalyst modifiers”, whose common purpose is to decrease the hyperactivity of the (Pd) metal. It is clear that, besides the use and consumption of toxic/expensive substances, drawbacks in terms of catalyst reuse and deactivation pose severe limitations in the utilization of this approach.

As it will be illustrated in the following sections, hydrogenation under continuous-flow conditions may represent a favourable alternative. Catalytic flow systems have shown to be extremely beneficial for carrying out chemical processes that are difficult to perform under batch conditions, e.g., involving reactive intermediates or competitive reactions [74–75]. Compared to batch setups, performing reactions under continuous flow allows a fine tuning of the contact time between intermediates and catalytic active phase, which may result in improved selectivity, with no need of additives [76].

### Reactor and catalyst design

In contrast to unselective processes in the gas phase (e.g., for bulk chemicals production), whose fast interaction with the catalyst may ensure satisfactory conversions under the reaction conditions, selective liquid phase flow processes for the fine-chemicals synthesis, including partial hydrogenations, usually requires a more intimate contact with the heterogeneous catalyst to be efficient. One example is the so-called "confinement effect" found in mesoporous catalytic materials [77–78].

**Figure 1:**
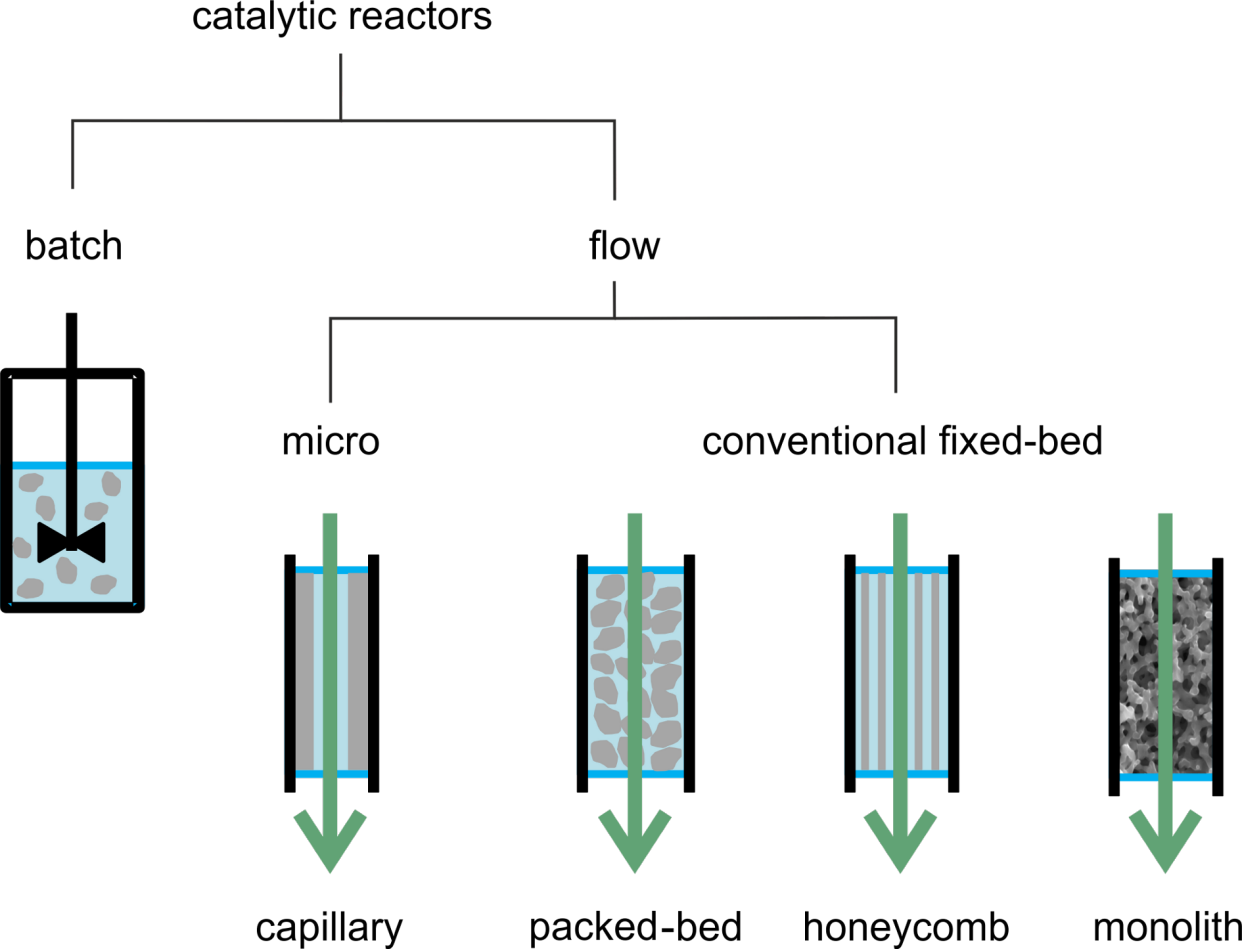
Schematic representation of most common reactor types for batch and continuous-flow partial hydrogenation of alkynes. Particles, layer or body of the catalyst in grey.

Catalytic hydrogenations are conveniently achieved under flow using fixed bed devices, wherein the size of the inner diameter of reactor channels distinguishes between micro (10–500 μm) or mesofluidic (500 μm up to several mm) reactors [79–80]. This size range may allow for the production of mg to tens of tons of target compound per year [81]. Despite the several possible reactor arrangements and catalyst morphologies falling within the above classification [82–83], herein we decided to break down the systems according to the main types reported in the literature for the catalytic partial hydrogenation reaction of alkynes, i.e., capillary, packed-bed, honeycomb and monolithic reactors. A schematic representation of these reactors is shown in Figure 1. Other reactor types (e.g., fluidized bed reactor, wherein the solid catalyst is suspended in a fluid) have not been reported for liquid-phase alkyne hydrogenations and will not be discussed.

**Figure 2:**
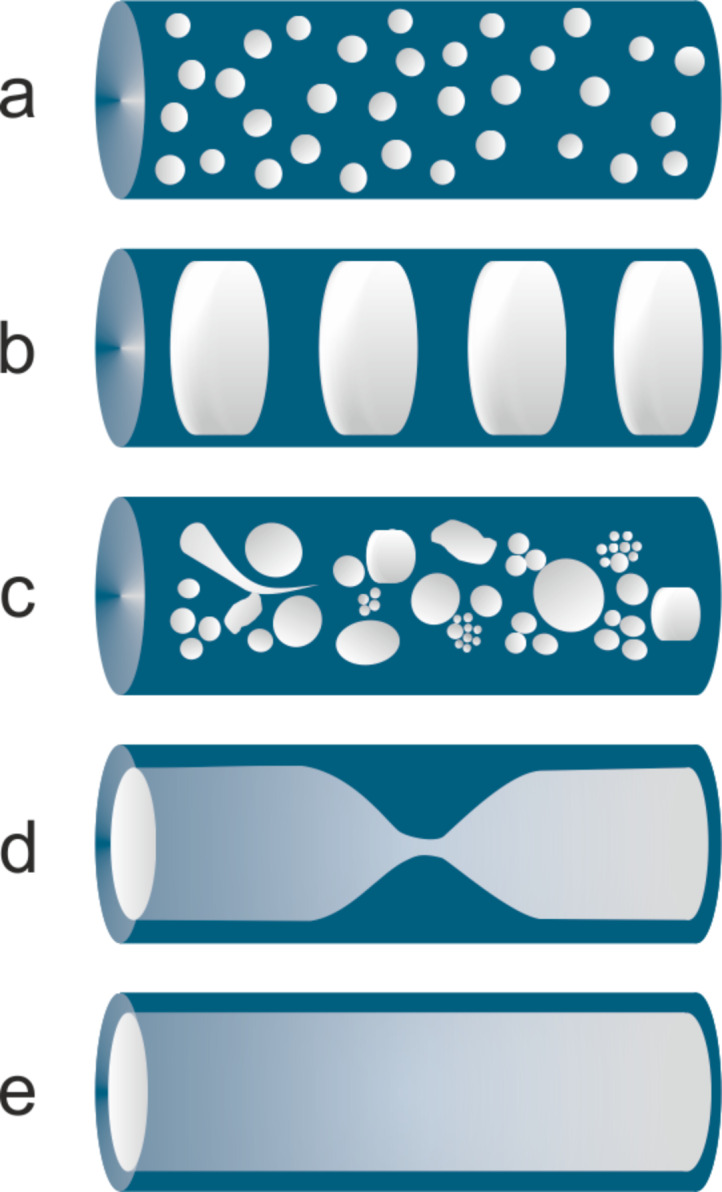
Schematic representation of flow regimes in microchannels; (a) bubbly flow, (b) slug/Taylor or segmented flow, (c) churn flow, (d) slug/annular flow, (e) annular flow.

Capillary reactors (10–1000 μm internal diameter, 0.1–30 m length) are routinely used in the lab-scale synthesis due to the ease of operations, negligible heat effects and fast reactants mixing [84–85]. Issues may arise from miniaturization of the catalysts, where the most common approach is to immobilize the catalyst onto the inner wall of the capillary support (catalytic wall reactors) [86–87] or to pack the powdered catalyst into the microchannels [88], the latter strategy being prone to significant pressure drops due to either the swelling or the size of the catalyst. It is important mentioning that different flow regimes can be attained in miniaturized channels, depending on the gas and liquid rates, as these may affect both conversion and selectivity of the process (Figure 2).

Packed-bed reactors are among the most used systems since they are relatively simple to handle, easy to operate and can accommodate beds with a broad range of physical dimensions and shapes. Commonly used packed catalysts consist of metal nanoparticles (MNP) immobilized onto a variety of porous solid supports, either pellets or powders of various grain size (in the range μm to mm). The preferred choice are mesoporous supports (2–50 nm cavity size), due to the enhanced contact with the reagents because of the high surface area [89–90] and the effective “steric” stabilization of the embedded metal nanoparticle catalysts [91–92]. However, mesoporous catalysts may suffer from pore clogging, active sites accessibility, mass transfer limitations, and lack of reproducibility. Additional stabilization of MNP can be also achieved either by: the “electrostatic” effect of charged functional groups grafted to the support, a common strategy in gel-type resins (e.g., sulfonic resins) [93]; the strong metal–support interactions, particularly for inorganic oxide materials, e.g., TiO_2_ [94–95]. Besides contributing to catalyst resistance by hampering the loss and the size increase of active sites, MNP stabilization is a key factor to limit the amount of metal leached in solution, an issue of utmost importance for the reduction of metal residues in the food and pharmaceuticals manufacture industry [96]. The choice of appropriate support materials is therefore critical to this purpose. Alternative strategies to reduce metal contamination include the use of metal scavengers, usually in the form of a downstream located cartridge [97].

Honeycomb (or foam) catalysts consist of inert carrier materials with millimetre size parallel channels (or cavities) obtained by extrusion, onto which a catalytically active layer is deposited, usually a porous inorganic oxide bonded to the support surface and containing precious metal sites (washcoat) [98–99]. They are largely reported in the chemical engineering literature for gas-phase, unselective thermal processes [100–101].

A monolith is “a shaped, fabricated intractable article with a homogeneous microstructure that does not exhibit any structural components distinguishable by optical microscopy” [102]. According to this definition, honeycombs do not fall within this category. In the recent years, porous monoliths have attracted considerable interest in several flow-through applications for the fine chemistry, including chromatography and catalysis [103–104]. Monolithic reactors may surpass most drawbacks typical of packed-bed systems, including high pressure drops, low contacting efficiency, large distribution of residence times, formation of hot-spots or stagnation zones, which results in poorly controlled fluid dynamics, hence in low catalyst productivity and selectivity [105–106]. Particularly, monoliths featuring a 3D isotropic, hierarchically porous network of narrowly size distributed, interconnected macropores (1–30 μm) and mesopores within the struts (6–50 nm) have shown a unique hydrodynamic behavior in the liquid phase [107–108], which addresses the need of both efficient processing (within small pores) and effective mass transport (by macropores) [109–110]. This kind of monolith obtained by spinodal decomposition joins the advantages of high surface area typical of mesoporous material, spanning from 200 to 1200 m^2^ g^−1^ [111] with a high permeability typical of macropores, which results in a very efficient mass transfer [112]. According to Darcy’s law, describing the flow of a fluid through a porous medium, hierarchically porous monoliths show a very low pressure drop Δ*p* per unit reactor length *L* (Δ*p*/*L* = (μ*v*)/*k*, μ viscosity; *v* linear velocity), thanks to the high permeability coefficient *k* > 0.25 μm^2^, which is proportional to the macroporous size *D*^2^ [113]. The catalytic performance of these monoliths has been compared in continuous flow as a single piece or packed-bed (ground monolith 60–120 μm) and in batch arrangements. The better productivity was clearly demonstrated for the entire monolith under flow in the hydrogenation reaction of cyclohexene, resulting in turnover frequencies of 1673, 1131 and 932 h^−1^ and space-time-yields of 4.02, 0.95 and 0.01 kg_product_ L_reactor_^−1^ h^−1^, respectively [109]. An analogous permeability was observed under flow for 1D nanostructured support materials (vide infra).

**Figure 3:**
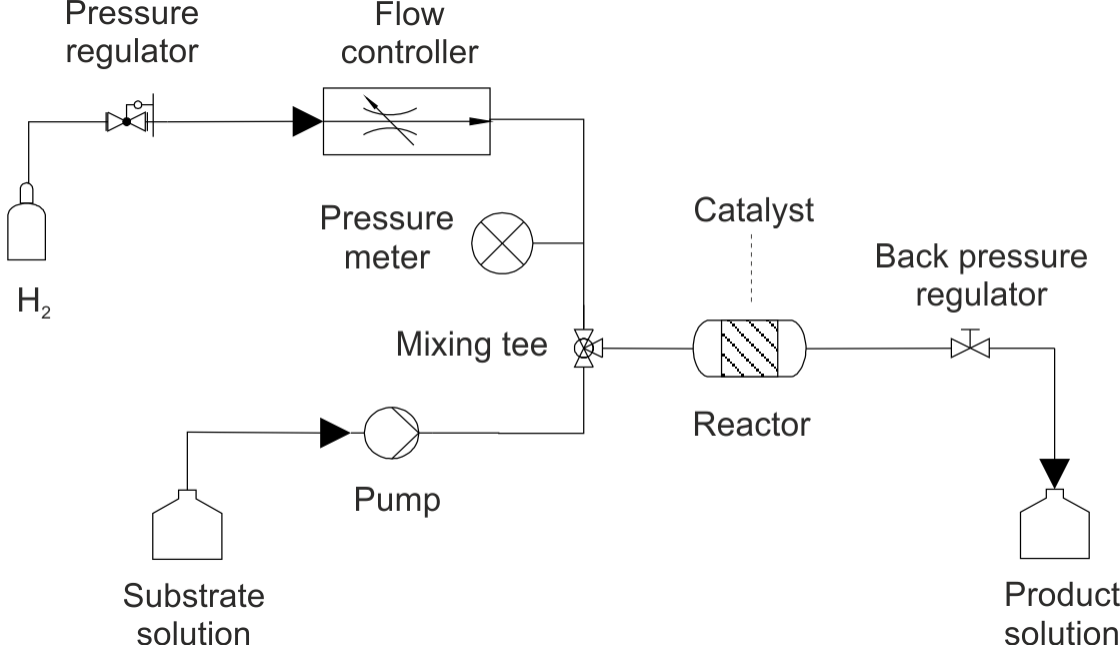
Sketch of typical continuous flow apparatus for liquid-phase catalytic alkynes hydrogenation reactions.

A typical equipment for liquid-phase continuous-flow hydrogenations is sketched in Figure 3. Usually, concurrent, controlled flows of substrate solution and H_2_ gas are allowed to flow through the catalytic reactor. Reaction products are collected at the reactor outlet. Typical residence times for alkynes partial hydrogenations, which defines the amount of time that the reaction mixture spends inside the reactor (the volume of the reactor divided by the volumetric flow rate) [114], are in the range 10–1000 s, corresponding to 5 μL/min (for capillary reactors) up to 3 mL/min of substrate solution flow. Hydrogen flow rates (and pressures) are adjusted to have typical H_2_: substrate molar ratios inside the reactor in the range of 1–30.

### Hydrogenation of terminal alkynes

**Scheme 2:**
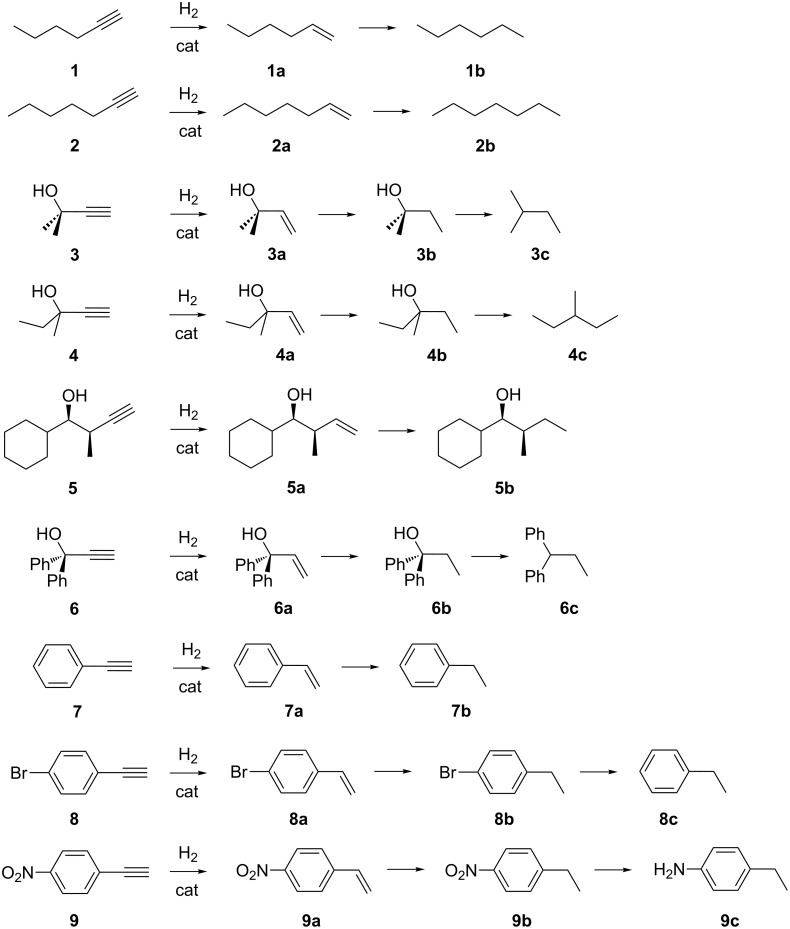
Hydrogenation reactions of terminal alkynes with potential products and labelling scheme.

Various terminal alkynes have been hydrogenated under continuous-flow conditions using supported catalysts. The substrates and the commonly observed products with the labelling adopted in the present review are shown in Scheme 2. Representative data are summarized in Table 1, in which conversions are indicated and catalysts’ efficiencies are expressed in terms of selectivity, yield of indicated product, mass productivity (mol_product_ g_metal_^−1^ h^−1^) and space-time-yield (STY) [115]. Whenever available, the best compromise results between conversion and selectivity are reported.

**Table 1:** Representative continuous flow catalytic processes for liquid-phase partial hydrogenation of terminal alkynes.

entry	alkyne	reactor^a^	catalyst	*T* (K)	conv.^b^ (%)	selectivity^c^ (%)	yield^d^ (%)	prod.^d,e^ (mol g_M_^−1^ h^−1^)	STY^d^ (kg L^−1^ h^−1^)	ref.

1	**1**	PB	5% Pd(Pb)@CaCO_3_	293	90^f^	94	84.6	2.9	1.65	[119]
2	**1**	PB	16.2% CeO_2_@TiO_2_	413	97^g,h^	100	97.0	1.1	18.86	[119]
3	**1**	PB	0.6% Pd(HHDMA)@C	293	30^f^	97	29.1	0.8	0.13	[121]
4	**1**	PB	0.5% Pd(HHDMA)@TiS	293	30^f^	96	28.8	0.9	0.13	[121]
5	**1**	PB	1.0% Pd@Al_2_O_3_	293	30^f^	67	20.1	0.3	0.09	[121]
6	**1**	PB	0.5% Pd@mpg-C3N4	343	–^i,j^	90	–^i^	13.3	–^i^	[122]
7	**1**	PB	1.3% Ag@SiO_2_	373	30^k^	95	28.5	0.1	0.13	[123]
8	**1**	PB	1% Au@TiO_2_	373	40^k^	95	38.0	0.1	0.17	[123]
9	**1**	PB	1% Ag@TCM-mpg-C3N4	303	–^i,k^	100	–^i^	0.9	0.56	[124]
										
10	**3**	C	1% Pd_25_Zn_75_@TiO_2_	333	>99^l^	90	89.4	5.3	<0.01	[131]
11	**3**	M	0.67% Pd@MonoBor	294	92^l^	94	86.4	16.2	1.52	[136]
12	**3**	PB	0.5% Pd@TiNT	294	75^l^	83	62.2	82.3	7.36	[137]
13	**3**	PB	0.6% Pd(HHDMA)@C	293	30^f^	100	30.0	0.9	0.17	[121]
14	**3**	PB	0.5% Pd(HHDMA)@TiS	293	30^f^	96	28.8	1.0	0.16	[121]
15	**3**	PB	5% Pd(Pb)@CaCO_3_	293	30^f^	83	24.9	0.1	0.14	[123]
16	**3**	PB	1.3% Ag@SiO_2_	373	30^k^	100	30.0	0.1	0.17	[123]
17	**3**	PB	1% Au@TiO_2_	373	30^k^	100	30.0	0.1	0.17	[123]
18	**3**	PB	0.5% Pd@mpg-C3N4	343	–^i,j^	90	–^i^	13.2	–^i^	[122]
19	**3**	PB	0.1% Pd@NKZPDB-5	294	>99^l^	60	59.4	5.8	0.17	[141]
										
20	**4**	C	0.02 wt % Pd@Al_2_O_3_	293	94^f^	83	78.0	–^i^	–^i^	[142]
										
21	**5**	PB	5% Pd(Pb)@CaCO_3_	298	95^k^	100	95	–^i^	–^i^	[143]
										
22	**6**	M	0.67% Pd@MonoBor	294	85^l^	84	71.3	0.9	0.20	[136]
23	**6**	PB	0.5% Pd@TiNT	294	78^l^	89	69.4	20.0	4.37	[137]
24	**6**	PB	5% Pd(Pb)@CaCO_3_	293	30^f^	100	30.0	0.1	0.39	[123]
25	**6**	PB	16% CeO_2_@TiO_2_	413	51^g^	100	51.0	<0.1	0.29	[119]
26	**6**	PB	1.3% Ag@SiO_2_	373	30^k^	96	28.8	0.1	0.38	[123]
27	**6**	PB	1% Au@TiO_2_	373	30^k^	100	30.0	0.1	0.39	[123]
										
28	**7**	PB	16% CeO_2_@TiO_2_	413	100^g^	100	100.0	<0.1	0.25	[119]
29	**7**	M	0.67% Pd@MonoBor	294	98^l^	96	93.7	2.9	0.33	[136]
30	**7**	PB	0.5% Pd@TiNT	294	83^l^	82	68.1	6.5	0.71	[137]
31	**7**	PB	5% Pd(Pb)@CaCO_3_	293	30^f^	98	29.4	0.1	0.17	[123]
32	**7**	PB	1.3% Ag@SiO_2_	373	30^k^	100	30.0	0.1	0.18	[123]
33	**7**	PB	1% Au@TiO_2_	373	30^k^	96	28.8	0.1	0.17	[123]
34	**7**	PB	0.3% Au@TiO_2_	333	99^m^	100	99	1.9	0.67	[151]

^a^Reactor type: C, capillary; PB, packed-bed; M, monolithic. ^b^Substrate conversion. ^c^Selectivity to the alkene product, e.g., **1a** / (**1a** + **1b**). ^d^Calculated on the basis of the alkene product. ^e^Calculated on bulk supported metal loading. ^f^1 bar H_2_. ^g^90 bar H_2_. ^h^Solvent-free. ^i^Not available. ^j^5 bar H_2_. ^k^10 bar H_2_. ^l^1 – 2.7 bar H_2_. ^m^No H_2_ pressure specified.

#### 1-Hexyne and 1-heptyne

The partial hydrogenation of 1-hexyne (**1**) produces 1-hexene (**1a**), one of the most commercially important linear α-olefins used in copolymerization processes [116–117]. High density polyethylene (HDPE) and linear low density polyethylene (LLDPE) contain approximately 2–4% and 8–10% of **1a**, respectively [118]. 1-Hexene can be produced in ca. 91% yield under batch conditions using bimetallic Pd (4 wt %)–Cu (2 wt %) catalysts immobilized onto silica (298 K, 1 bar H_2_) [65].

**Figure 4:**
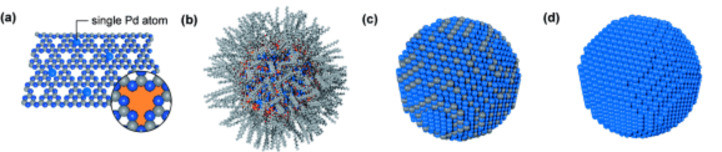
Structure of Pd@mpg-C3N4 (a), Pd(HHDMA)@C (b), Pd(Pb)@CaCO_3_ (c) and Pd@Al_2_O_3_ (d) catalysts. The structures depict the increasing size of the active ensemble, from a single Pd atom (a) to a bare PdNP of approximately 800 atoms (d). The inset in (a) shows a characteristic six-fold cavity (orange) in the carbon nitride structure. C light grey, N dark blue, O red, Pb grey, Pd light blue. Adapted with permission from [122] , © 2015 John Wiley and Sons.

1-Hexyne was used as benchmark substrate to compare the efficiency of various packed-bed hydrogenation catalysts under continuous flow. Outstanding **1a** yields were obtained either using the Lindlar catalyst (84.6%) under smooth reaction conditions (298 K, 1 bar H_2_, Table 1, entry 1) or 16.2 wt % CeO_2_@TiO_2_ (97%), the latter resulting in a very high STY for **1a** (18.86 kg L^−1^ h^−1^) under solvent-free conditions (Table 1, entry 2) [119]. Use of cerium oxide is certainly advantageous in terms of catalyst cost, however, it requires much stronger reaction conditions to afford conversions analogous to that of Pd (413 K, 90 bar H_2_). Comparable selectivities, although at lower conversion level, were reported using ligand-modified Pd catalysts, namely hexadecyl-2-hydroxyethyl-dimethylammonium dihydrogen phosphate (HHDMA), commercially available under the name NanoSelect^TM^ [120]. Low-content, colloidal HHDMA-palladium catalysts onto on activated carbon (Table 1, entry 3) or titanium silicate (Table 1, entry 4) provided selectivities for **1a** of 97% and 96% respectively. As expected, “bare” 1% Pd@Al_2_O_3_ (Table 1, entry 5) showed to be poorly selective (67%), resulting in considerable amounts of oligomers and over-hydrogenated product **1b** [121]. The performance of the above systems was compared with that of a single-site catalyst based on Pd atoms confined into the “six-fold cavities” of a mesoporous polymeric graphitic carbon nitride (mpg-C3N4, Figure 4a) [122]. The catalyst showed the highest productivity in the series (13.3 mol**_1a_** g_Pd_^−1^ h^−1^), under fairly mild conditions (343 K, 5 bar H_2_) (Table 1, entry 6), which was attributed to the facile hydrogen activation and alkyne adsorption on the atomically dispersed Pd sites. Electrostatic stabilization of Pd atoms was ascribed to a strong interaction with the nitrogen-coordinating species on the basis of DFT calculations. Effective stabilization also prevented site aggregation, resulting in pretty constant catalytic activity over a 20 h time-on-stream.

As an alternative to poisoned PdNP, use of other noble metals was also explored, although with lower catalysts efficiency. For instance, Ag@SiO_2_ (Table 1, entry 7) and Au@TiO_2_ (Table 1, entry 8) provided **1a** with modest yields and productivities under more severe reaction conditions compared to Pd (373 K, 10 bar H_2_) [123]. Interestingly, the productivity of these systems could be significantly improved by adopting the same atomic level dispersion approach above described for palladium. Thus, Ag onto tricyanomethanide doped mpg-C3N4 provided comparatively much higher reaction rate for **1a** (0.9 mol g_Pd_^−1^ h^−1^) (Table 1, entry 9) [124], that confirms the effectiveness of the strategy.

The continuous flow partial hydrogenation of 1-heptyne (**2**) to 1-heptene (**2a**), an additive for lubricants and a surfactant [125], was also reported using packed 2% Pd@Al_2_O_3_ catalyst, showing 49% selectivity at 81% conversion under room temperature and 1 bar H_2_ (STY for **2a**: 0.12 kg L^−1^ h^−1^), with no detectable signs of deactivation over 6 h reaction time [126].

#### 2-Methyl-3-butyn-2-ol and 3-methyl-1-pentyn-3-ol

The catalytic partial hydrogenation reaction of 2-methyl-3-butyn-2-ol (**3**) is an in-depth studied process, mainly because the alkene product (**3a**) is an important intermediate for the industrial synthesis of vitamins (A, E), as well as a variety perfumes [127–128]. The current manufacturing process is based on the Lindlar or other Pd-based heterogeneous catalysts under batch conditions. Yields of the desired product are in the range of 95–97%, however, with fast catalyst deactivation due to degradation of the support or sintering of metal particles [129–130].

Reports exist on the partial hydrogenation of **3** under continuous flow. Best results in terms of alkene yield (89%) were reported for a capillary microreactor (10 m length, 250 μm i.d., 110 nm film thickness) operating under annular two-phase flow regime, and whose inner walls were coated with a bimetallic Pd_25_Zn_75_ catalyst onto mesoporous TiO_2_ (Table 1, entry 10). Selectivity could be further improved (97%) by addition of harmful pyridine [131]. The rather systematic kinetic study showed the significant selectivity increase (ca. 10%) by addition of Zn to the monometallic Pd catalysts, even at high substrate concentrations (up to 0.45 M). Selectivity enhancement could be attributed to a Pd site-isolation effect, similar to that found in Lindlar catalysts, which reduces the number of multiple interactions of the adsorbed intermediate alkene with active hydrogen species. It must be noted, however, the low STY value provided by this reactor type (less than 0.01 kg L^−1^ h^−1^) due to the low substrate fed allowed (max. 14 μL min^−1^). A lower selectivity was observed for the corresponding batch setup that was justified in terms of a slightly different concentration of active sites in the catalysts, as a result of the different preparation procedures. Similar findings were observed for an analogous Pd@TiO_2_ capillary reactor (annular flow), which showed ca. 15% higher selectivity compared to the corresponding batch system, although under slightly different reaction conditions [132]. The result was attributed to the shorter contact time between reagent and catalyst in that case.

**Figure 5:**
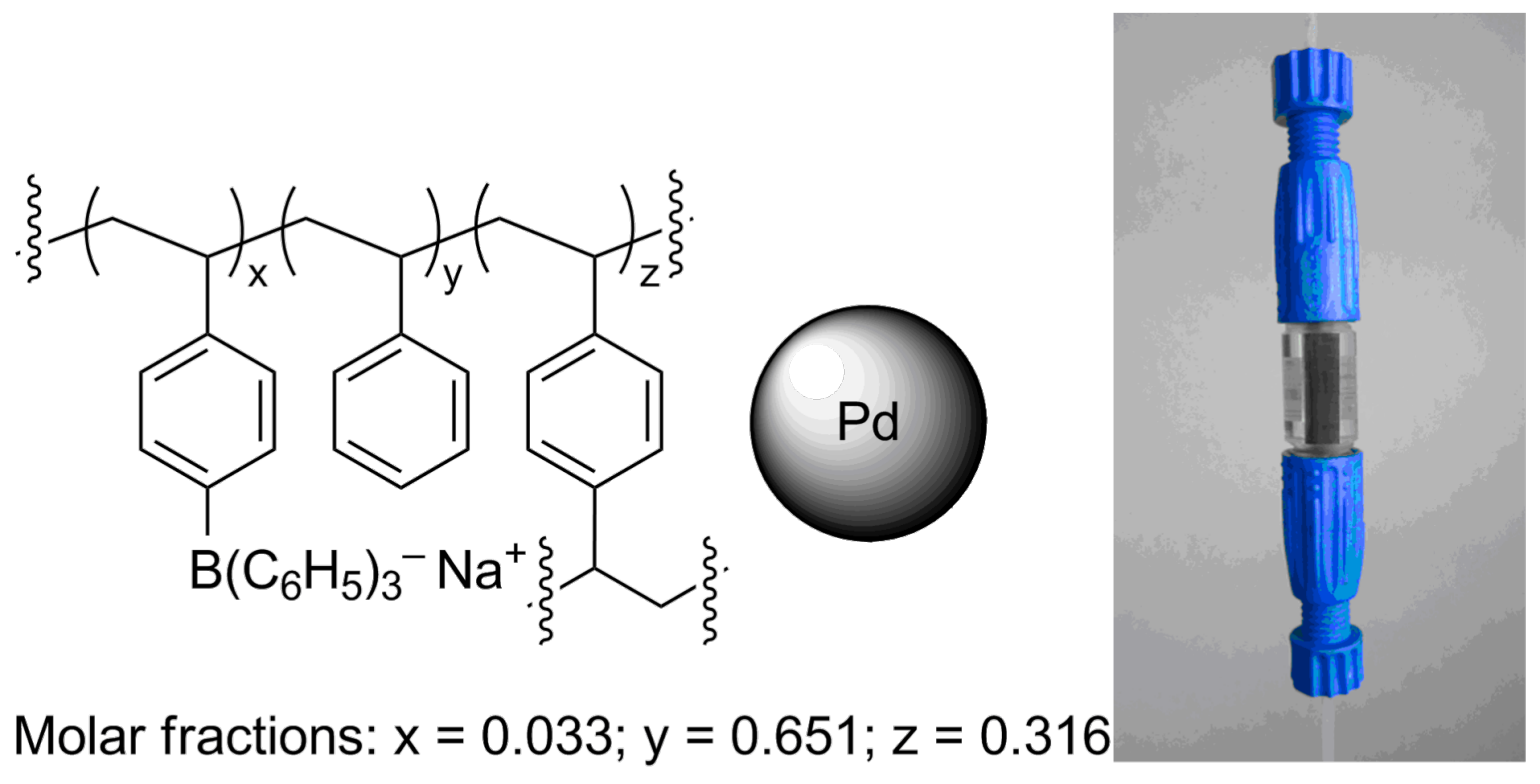
Sketch of composition (left) and optical image of Pd@MonoBor monolithic reactor (right). Adapted with permission from [136], © 2013 Elsevier.

**Figure 6:**
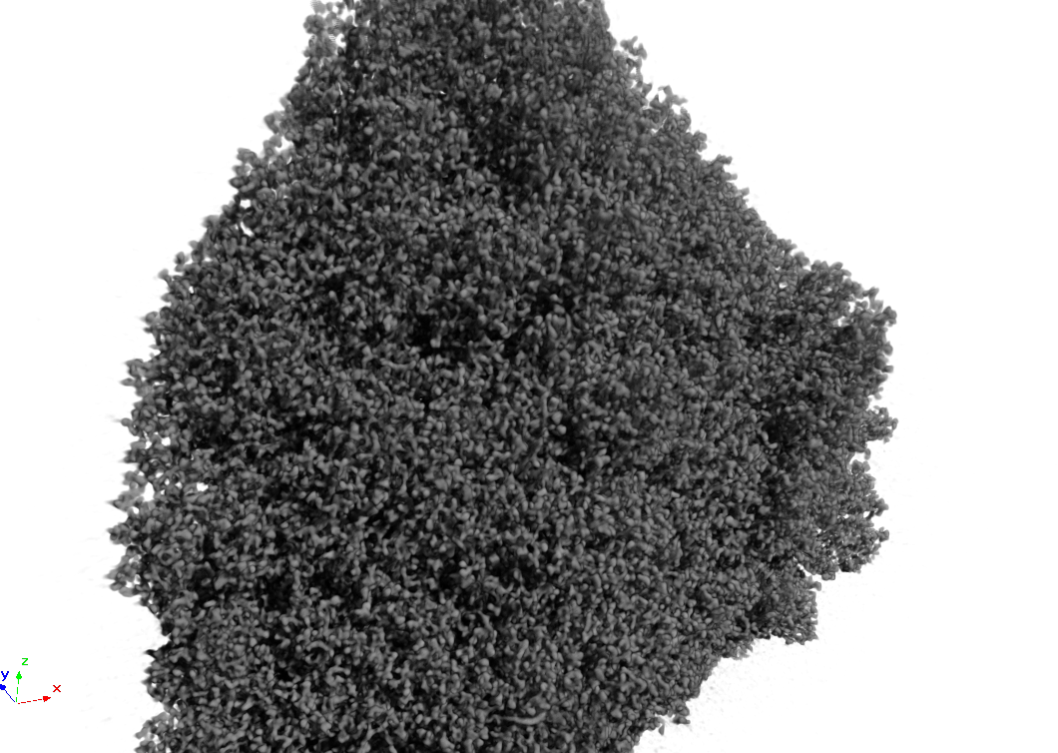
X-ray tomography 3D-reconstruction image of MonoBor [133]. Unpublished image from the authors.

In a different approach, PdNP were immobilized onto an open-cell macroporous (10 μm pore size), polymeric borate monolith, that was grown in situ within the walls of a commercial, tubular glass reactor (MonoBor, reactor volume 176 μL, Figure 5) [133]. The monolithic support was specifically designed to allow for the immobilization of Pd particles at non-coordinating borate sites within a rigid, highly-cross linked solid matrix (Figure 6) [134–135]. Under mild conditions (294 K, 1.4 bar H_2_), alkene **3a** was obtained with good yield (93.9% sel. at 92% conv.) and productivity (1.52 kg L^−1^ h^−1^) (Table 1, entry 11) [136]. The latter value was attributed to the macroporosity and to the poor swelling volume of the support material, which allow for high flow rates to be attained with low back-pressure evolution (methanol solution 0.1 M, 0.6 mL min^−1^, H_2_ pressure drop 0.4 bar).

**Figure 7:**
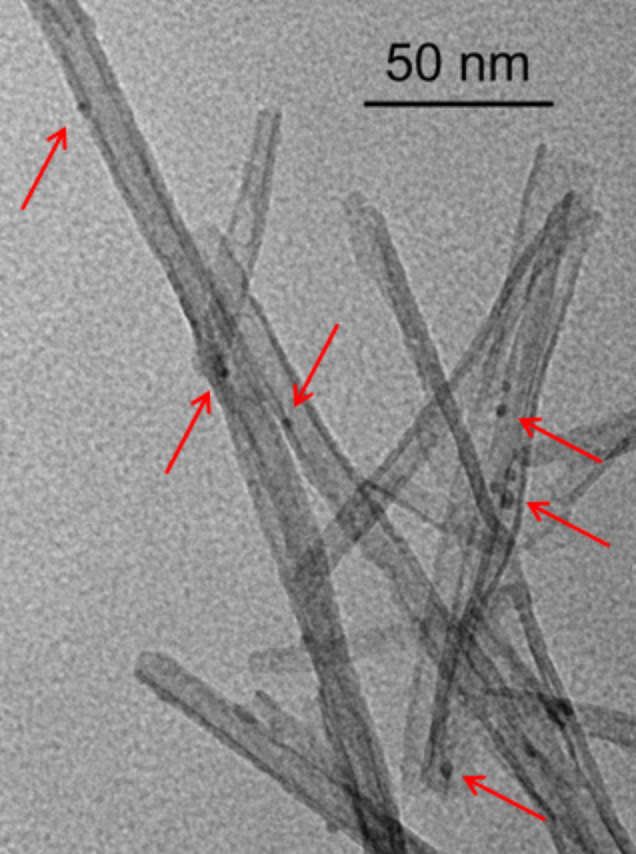
Representative TEM image of titanate nanotubes with immobilized PdNP (arrows). Adapted with permission from [137], © 2016 John Wiley and Sons.

The highest productivity in the continuous partial hydrogenation of **3** (82.3 mol g_Pd_^−1^ h^−1^, Table 1, entry 12), was obtained using a packed-bed catalyst based on PdNP onto the outer surface of titanate nanotubes (Figure 7), that was justified in terms of both accessibility of Pd sites and high permeability of the packed 1D tubular material (weight hourly space velocity, g_substrate_ g_catalyst_^−1^ h^−1^ ca. 11088 h^−1^) [137–138]. In fact, much lower efficiency was observed under analogous flow conditions using Pd immobilized into the pores of conventional mesoporous powder titania.

Analogous benefits in liquid phase flow operations were obtained by using nanostructured fibrous materials, that was attributed to the enhanced mass transfer of the one-dimensional packed support [139]. It is worth noticing that this result was obtained as a consequence of the so-called “Rational Catalyst Design” approach [140], applied to the hydrogenation of 2-methyl-3-butyn-2-ol as a case study [29]. In this approach, an optimized catalyst was designed by the integration of the catalyst performances at increasing length scales, from the nanoscale (active metal nanoparticles), to the mesoscale (support) and macroscale (reactor). Thus, after identification of the optimal shape and size of PdNP for the hydrogenation of **3**, the ex-situ particles were deposited onto a ZnO/Sintered Metal Fibers support, having selected this material for its excellent mass transfer properties. The catalyst was then integrated into a bubble column flow reactor with staged catalytic layers, showing two order magnitude higher productivity compared to Lindlar catalyst [141].

Low to moderate yields and productivities for **3a** were reported by using conventional packed-bed catalysts, either HHDMA-modified (Table 1, entries 13, 14) [121], Lindlar (Table 1, entry 15) or non-palladium based (Table 1, entries 16, 17) [123]. As above discussed for **1**, single-atom catalysts resulted in high productivity, however, direct comparison of product yield with other systems is not possible due to insufficient experimental data (Table 1, entry 18) [122]. PdNP onto hybrid zirconia/polyvinyl alcohol matrix (NKZPD) were also described, providing **3a** in moderate selectivity (60%) at full conversion and mild conditions (Table 1, entry 19) [142].

The partial hydrogenation reaction of the parent alkyne 3-methyl-1-pentyn-3-ol (**4**) was also reported under continuous flow. Similarly to what described above for **1** and **3**, the hydrogenation of **4** to **4a** was investigated in detail by comparing the selectivity of diverse Pd packed-bed catalysts at the same substrate conversion level (30%). The study confirmed the efficiency of the catalysts to decrease in the order Pd(HHDMA)@C > Pd(HHDMA)@TiS > Lindlar > Pd@Al_2_O_3_ (from 100 to 67%) [121]. This selectivity trend was explained in terms of both adsorption mode on and relative accessibility to Pd active sites, depending on surface potentials and hindrance of modifiers, on the basis of density functional theory and molecular dynamics calculations. The rationale was summarized in the so-called thermodynamic selectivity concept, that is “a selective catalyst involves strong adsorption of the alkyne and a low stability to the adsorbed alkene” [121]. In bare Pd catalysts, such as Pd@Al_2_O_3_, or in alloyed Pd catalysts, such as Lindlar, the intermediate alkene is strongly adsorbed on Pd surface (exothermic process), thereby favouring further reaction with H_2_ and reducing selectivity. In bulky ligand-modified catalysts, such as Pd(HHDMA)@C, the adsorption process is slightly endothermic and selectivity is enhanced.

A high yield of **4a** (78%) was also obtained by means of a capillary microreactor consisting in a mesoporous Al_2_O_3_-coated commercial fused-silica column with embedded PdNP (530 μm i.d., 25 cm length, 6 μm thick layer) (Table 1, entry 20) [143]. Experiments were performed in a segmented flow regime (H_2_ gas/ethanol solution), so that the flow pattern enhanced the contact with the catalyst on the wall and minimize diffusion limits (Figure 2). Without bubbles, the yield of **4a** would have been ca. 57% at the same residence time. The catalyst was used for weeks without significant deactivation.

#### 1-Cyclohexyl-2-methyl-3-butyn-1-ol

In the course of their studies on diastereoselective chain-elongation reactions, Ley and Baxendale reported the hydrogenation of (1*R*,2*R*)-1-cyclohexyl-2-methyl-3-butyn-1-ol (**5**), where the partial reduction of the triple bond is achieved in the presence of stereogenic centres [144]. The alkene **5a** was obtained in 95% yield, without compromising the starting diastereomeric ratio (4.3:1), using the Lindlar catalyst packed into a commercial H-Cube^®^ apparatus under mild hydrogenation conditions (298 K, 10 bar H_2_, Table 1, entry 21).

#### 1,1-Diphenyl-2-propyn-1-ol

**Figure 8:**
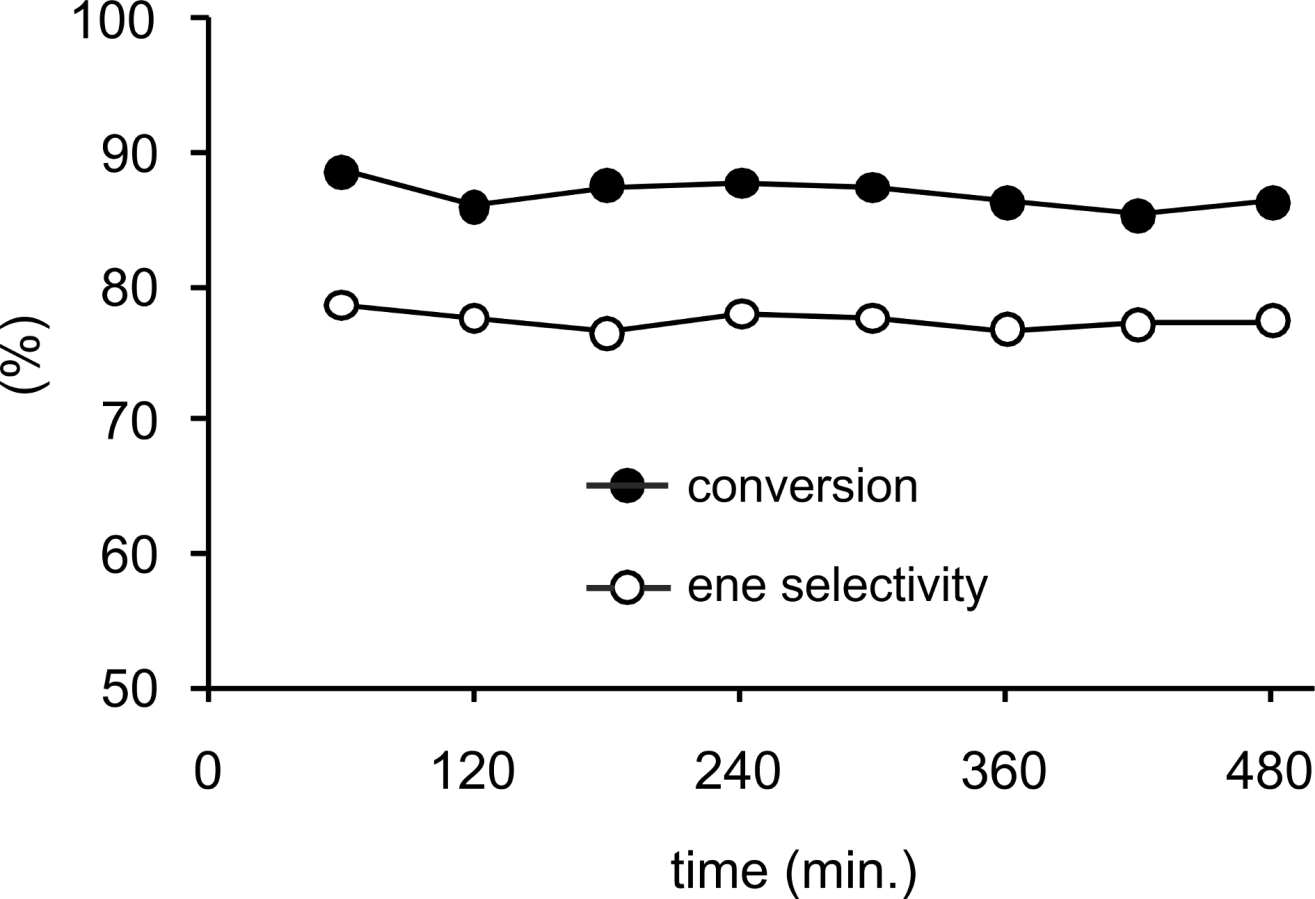
Conversion and selectivity vs. time-on-stream for the continuous-flow hydrogenation of **6** over Pd@MonoBor catalyst (methanol solution 0.16 mL min^−1^, H_2_ 1.00 mL min^−1^ @ 1.29 bar, rt). Reprinted with permission from [136], © 2013 Elsevier.

High yields of alkene **6a** were obtained by partial hydrogenation of 1,1-diphenyl-2-propyn-1-ol (**6**) using the monolithic Pd@MonoBor catalyst under smooth flow conditions (294 K, 84% selectivity at 85% conversion, Table 1, entry 22) [136]. Neither significant catalyst efficiency decay over 8 h reaction period was detected (Figure 8), nor an evidence for Pd leaching in solution was provided by ICP-OES throughout the reaction. This finding was justified by the effective electrostatic stabilization of PdNP by the charged -B(C_6_H_4_)^−^ groups onto the polymeric solid matrix. A small activity loss was attributed to poisoning by dimers and other byproducts adsorbed on the catalyst surface, as described for other batch Pd catalysts [145]. Under analogous conditions, Pd@TiNT gave **6a** in comparable yield (69.4%), although with remarkably higher productivity (for **6a**: 20.0 mol g_Pd_^−1^ h^−1^) (Table 1, entry 23) [137], as previously outlined in the case of alkyne **3**.

These latter results outperform those obtained at higher reaction temperatures using packed catalysts onto conventional supports, including Lindlar, CeO_2_@TiO_2_, Ag@SiO_2_ and Au@TiO_2_ (Table 1, entries 24–27), although the data are not properly comparable because they refer to significantly lower conversion levels (30–50%) [119,123]. The partial hydrogenation of **6** to **6a** under batch conditions was also described in 82–90% yield using phosphinated polymer incarcerated palladium catalysts [146].

The hydrogenation reaction of the bulky alkyne 1,1-diphenyl-2-propyn-1-ol (**6**) provides an interesting discussion example on how the relationship between catalyst architecture and substrate hindrance affects catalyst activity, even if not directly related to continuous flow operation conditions. It was proposed that ligand-modified surfaces, such as Pd(HHDMA)@C, are three-dimensional catalytic ensembles whose organic capping layer cannot be penetrated with ease by larger alkynes (Figure 4b) [121]. This justifies for the inactivity of Pd(HHDMA)-type catalysts toward the hydrogenation of **6**, while they are quite active in short-chain alkynes hydrogenation, e.g., **3** (Table 1, entries 13, 14) [123]. On the other hand, "naked" and Pb-poisoned palladium surfaces are 2D catalytic architectures (Figure 4c,d), which are amenable of alkynes adsorption irrespective of the chain length, thus resulting in high hydrogenation activity anyway. The selectivity is ruled by, e.g., site-isolation effects (Pd–Pd) in that case.

#### Phenylacetylene

The liquid-phase partial hydrogenation of phenylacetylene **7** was successfully achieved in the past using batch Pd catalysts (0.15–5% wt), with typical **7a** yields in the range of 60–70% [147–148]. More recently, a number of catalytic flow reactors were also described for this process. Best yields (94–100%) were achieved using either 16% CeO_2_@TiO_2_ (100%) [119] or Pd@MonoBor catalyst [136], under 90 bar H_2_ and 413 K or 1.3 bar H_2_ and 294 K, respectively (Table 1, entries 28, 29). As above reported for other substrates, best results in terms of productivity were provided by the monolithic and the titanate nanotubes-supported Pd catalysts (2.9–6.5 mol g_Pd_^−1^ h^−1^, Table 1, entries 29, 30) [137]. Lower performances were observed using packed-bed catalysts and conventional support materials [123], an amorphous Pd_81_Si_19_ alloy catalyst in supercritical CO_2_ (76% sel. at 91% conversion, 358 K) [149] or a capillary reactor (i.d. 250 μm) internally coated with Pd-doped mesoporous titania film (95% sel. at 30% conversion, 323 K) [150] (Table 1, entries 31–33).

The reduction of **7** to **7a** was also reported by transfer hydrogenation using formic acid / triethylamine as hydrogen source and packed Au@TiO_2_ (rutile) catalyst [151]. An outstanding 99.7% yield was achieved at 333 K, corresponding to a productivity for **7a** of 1.9 mol g_Pd_^−1^ h^−1^ (Table 1, entry 34). This value was considerably higher (ca. 40%) than that obtained for the batch-type reaction under identical conditions. The selectivity toward **7a** was retained during the continuous operations, while a progressive decrease of conversion from 99% to 85% was observed after 3 h time on stream, that was partially recovered by treatment with acetone.

#### 1-Bromo-4-ethynylbenzene and 1-ethynyl-4-nitrobenzene

The hydrogenations of **7**, 1-bromo-4-ethynylbenzene (**8**) and 1-ethynyl-4-nitrobenzene (**9**) were also reported with modest yields to **7a** (56%), (**8a**) (21%) and (**9a**) (21%), eventually with the addition of triethylamine, using a packed-bed multichannel catalytic reactor. The catalyst was based on PdNP onto tri-modal (micro, meso), hierarchical porous synthetic carbon [152]. No catalyst deactivation was detected over 5 hours continuous runs (333 K, 1 bar H_2_).

**Figure 9:**
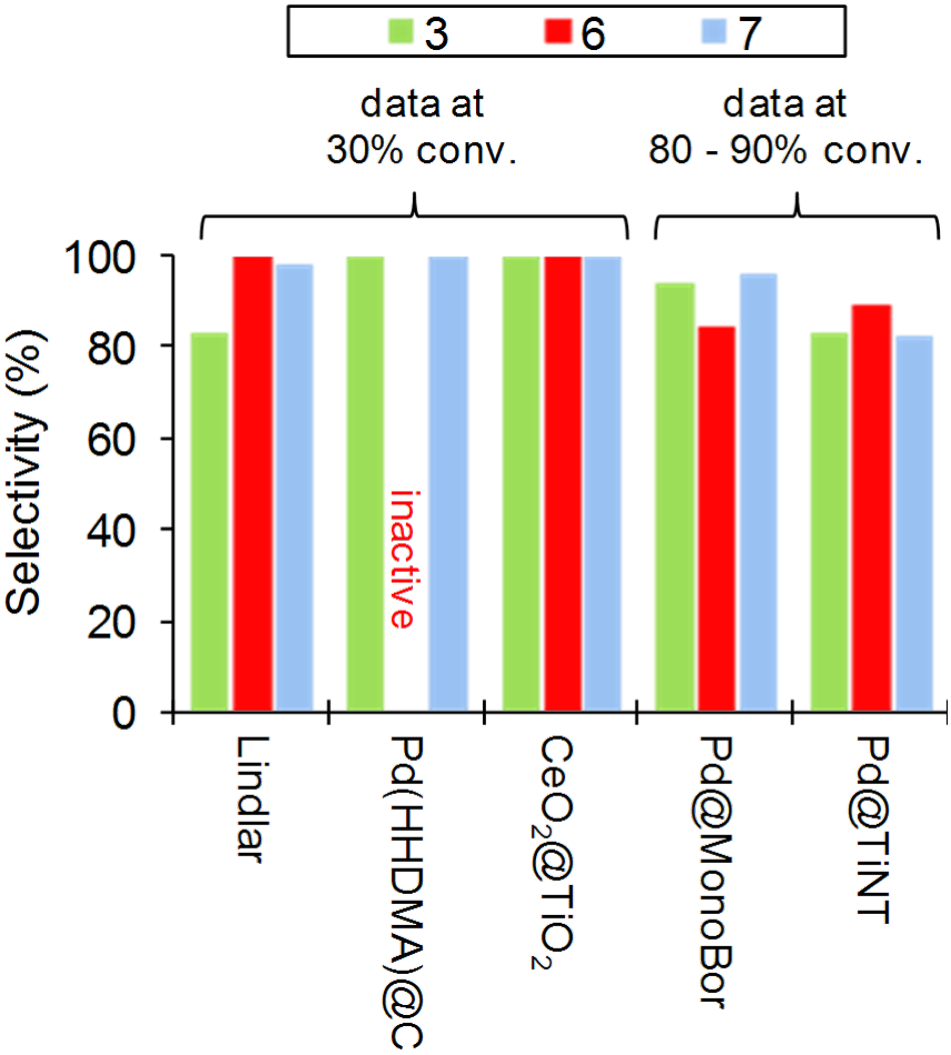
Continuous-flow hydrogenation of **3**, **6** and **7** over different catalytic reactor systems. Data from refs. [121,119,123,136–137].

A perusal of Table 1 shows that identification of the most versatile partial hydrogenation flow system for terminal alkynes, either catalyst or reactor, is prevented by significant substrate specificity, lack of experimental data or choice of parameter to be compared either selectivity, productivity or STY. As representative example for selected catalysts and substrates **3**, **6** and **7**, one can infer that CeO_2_@TiO_2_ usually provides better selectivity compared to other systems with strong substrate dependence (such as Pd(HHDMA)@C, see above discussion) (Figure 9). However, data are not available at the same conversion level, yet not directly comparable. Comparison in terms of, e.g., productivity is limited due to the same reason.

### Hydrogenation of internal alkynes

**Scheme 3:**
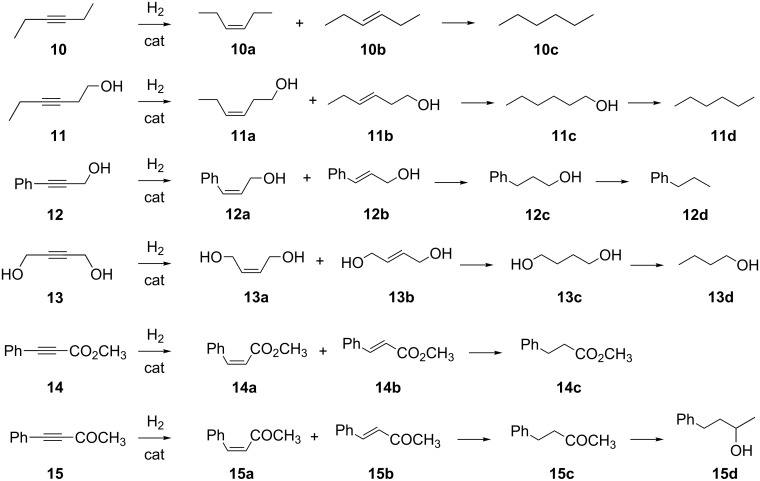
Hydrogenation reactions of internal alkynes with potential products and labelling scheme.

Compared to terminal alkynes, the partial hydrogenation reaction of internal alkynes is more challenging and intriguing owing to the stereoselectivity involved (usually *cis*) and to the large use of the products thereof in the fine-chemical industry. Most significant substrates examined in the literature under the continuous-flow catalysis conditions are reported in Scheme 3.

#### 3-Hexyne

Analogously to the earlier discussed hydrogenation of 1-hexyne (see above), the continuous flow, partial hydrogenation of 3-hexyne (**10**) to *cis*-3-hexene (**10a**) has been extensively examined by comparing the efficiency of various packed-bed supported catalysts [119,121,123]. In all cases, irrespective of the metal or the support, the catalysts yielded the *cis*-alkene product with selectivity ≥89%, with the exception Pd/Al_2_O_3_ (Table 2, entry 1), in line with the previous observation on the selectivity to 1-hexene. The trend regarding catalysts efficiency was also very similar. High yields of **10a** (>87%) were observed for the Lindlar and the TiO_2_-supported ceria catalysts, with a better productivity for the former (Table 2, entries 2, 3). All the other catalysts, including the HHDMA ligand modified-Pd one (Table 2, entries 4, 5) and the Ag and Au-based catalysts (Table 2, entries 7, 8) showed comparable selectivity, although at a lower conversion level. No data regarding the long-term catalysts stability were provided. Exceptionally high productivity was once again obtained for **10a** by the isolated Pd atoms catalyst Pd@mpg-C3N4 (11.3 mol g_Pd_^−1^ h^−1^, Table 2, entry 6) [123].

**Table 2:** Representative continuous flow catalytic processes for liquid-phase partial hydrogenation of internal alkynes.

entry	alkyne	reactor^a^	catalyst	*T* (K)	conv.^b^ (%)	selectivity		yield^e^ (%)	prod.^f,g^ (mol g_M_^−1^ h^−1^)	STY^f^ (kg L^−1^ h^−1^)	ref.

						ene^c^ (%)	*Z*/*E*^d^ (%)				

1	**10**	PB	1.0% Pd@Al_2_O_3_	293	30^h^	–^i^	53	15.9	0.3	0.07	[121]
2	**10**	PB	5% Pd(Pb)@CaCO_3_	293	93^h^	–^i^	94^j^	87.4	3.1	1.73	[119]
3	**10**	PB	16% CeO_2_@TiO_2_	413	93^k^	–^i^	100	93.0	< 0.1	0.18	[119]
4	**10**	PB	0.6% Pd(HHDMA)@C	293	30^h^	–^i^	97	29.1	0.8	0.13	[121]
5	**10**	PB	0.5% Pd(HHDMA)@TiS	293	30^h^	–^i^	100	30.0	0.9	0.14	[121]
6	**10**	PB	0.5% Pd@mpg-C3N4	343	–^i,l^	–^i^	90	–^i^	11.3	1.64	[122]
7	**10**	PB	1.3% Ag@SiO_2_	373	30^m^	–^i^	89	26.7	0.1	0.12	[123]
8	**10**	PB	1% Au@TiO_2_	373	30^m^	–^i^	94	28.2	0.1	0.13	[123]
											
9	**11**	M	0.67% Pd@MonoBor	294	> 99^n^	95	93	87.5	6.8	0.75	[136]
10	**11**	PB	0.5% Pd@TiNT	294	88^n^	94	93	76.9	40.6	4.24	[137]
11	**11**	PB	0.73% Pd@TiO_2_	294	84^n^	80	85	57.1	19.2	0.20	[137]
12	**11**	PB	1.2% Pd@SiO_2_	294	40^n^	87	93	32.4	7.1	1.43	[137]
13	**11**	PB	5% Pd(Pb)@CaCO_3_	294	99^n^	64	62	39.4	0.2	0.10	[136]
14	**11**	PB	1.25% Pd/Dowex-Li	294	75^n^	80	89	53.4	2.3	0.73	[161]
15	**11**	PB	5% Pd@C	294	94^n^	22	81	16.8	0.9	0.16	[140]
16	**11**	PB	0.1% Pd@NKZPDB-5	294	99^n^	83	83	68.2	11.4	0.43	[141]
17	**11**	M	1.3% Pd@SiO_2_ monolith	298	85^n^	80	80	54.4	0.5	0.17	[162]
18	**11**	M	0.2% Pd@TiO_2_ monolith	294	61^n^	63	87	33.7	1.8	0.51	[163]
											
19	**12**	M	0.67% Pd@MonoBor	294	96^n^	79	95	71.9	0.8	0.12	[136]
											
20	**13**	M	0.67% Pd@MonoBor	294	93^n^	75	100	70.2	3.3	0.32	[136]
21	**13**	HC	0.5% Pd@Al_2_O_3_	328	90^o^	99	100	89.8	0.7	–^i^	[170]
22	**13**	PB	0.5% Pd@Al_2_O_3_	328	90^o^	94	93	78.7	1.0	–^i^	[170]
23	**13**	PB	5% Pd@C	323	92^p^	100	97	89.6	–^i^	–^i^	[172]
											
24	**14**	M	0.67% Pd@MonoBor	294	90^n^	91	96	79.2	6.1	1.09	[136]
											
25	**15**	M	0.67% Pd@MonoBor	294	92^n^	93	50	42.8	1.1	0.17	[136]

^a^Reactor type: C, capillary; PB, packed-bed; HC, honeycomb; M, monolithic. ^b^Substrate conversion. ^c^Selectivity to the alkene product, e.g., (**11a** + **11b**)/(**11a** + **11b** + **11c** + **11d**). ^d^Selectivity to the *Z*-alkene product, e.g., **11a**/(**11a** + **11b**). ^e^Yield of *Z*-alkene. ^f^Calculated on the *Z*-alkene product. ^g^Calculated on bulk supported metal loading. ^h^1 bar H_2_. ^i^Not available. ^j^Selectivity calculated as **10a**/(**10a** + **10b** + **10c**). ^k^90 bar H_2_. ^l^5 bar H_2_. ^m^10 bar H_2_. ^n^1–2.7 bar H_2_ K. ^o^2 bar H_2_. ^p^10 bar H_2_.

#### 3-Hexyn-1-ol and 3-phenyl-2-propyn-1-ol

The *cis*-partial hydrogenation product of 3-hexyn-1-ol (**11**), the so-called leaf alcohol **11a**, is an important product widely used as fragrance or perfumes component [153–154]. It is industrially obtained with a production volume of 400 t/y in ca. 96% selectivity at 99% conversion by means of a conventional Lindlar-based batch process [155–156]. Several systems have been reported on the lab scale for the catalytic hydrogenation of **11** under continuous-flow conditions.

**Figure 10:**
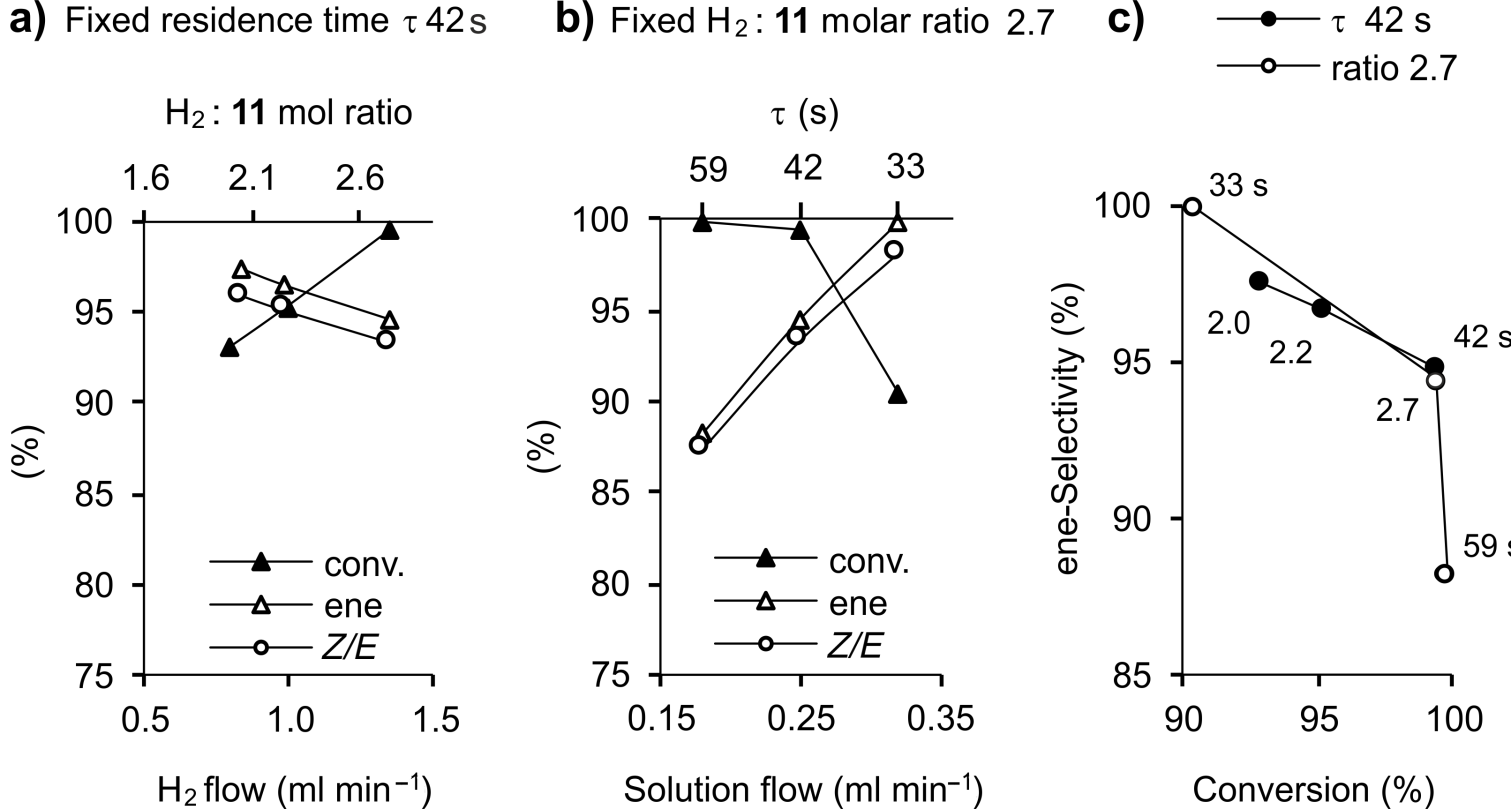
Continuous-flow hydrogenation of **11** over Pd@MonoBor catalyst. a) Conversion and selectivity as a function of H_2_ flow rate and H_2_:**11** molar ratio under fixed residence time τ 42 s (solution flow rate 0.25 mL min^−1^). b) Conversion and selectivity as a function of solution flow rate and residence time under fixed H_2_:**11** molar ratio 2.7. c) selectivity/conversion diagram at: ○ fixed H_2_:**11** ratio = 2.7 and residence time 33–59 s, • fixed residence time 42 s and H_2_:**11** ratio range 2.0–2.7. Data from ref. [136].

**Figure 11:**
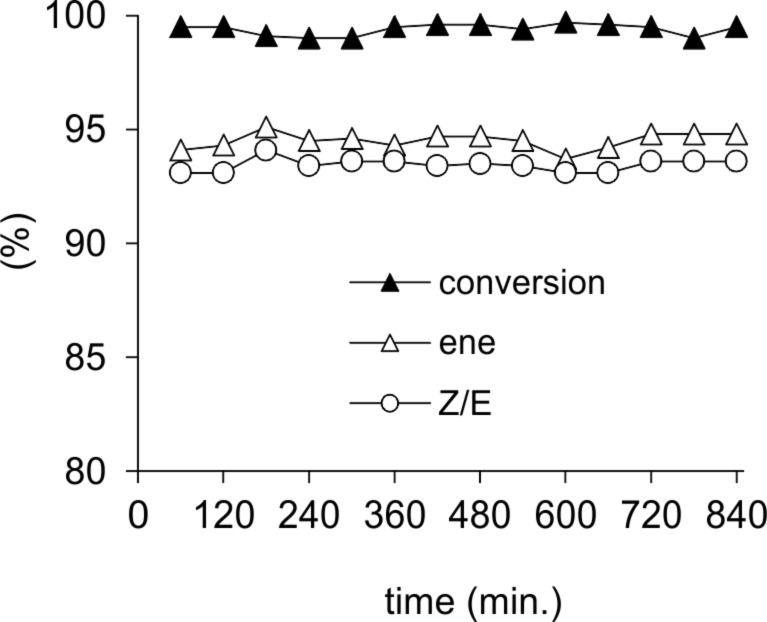
Conversion and selectivity vs time-on-stream for the continuous-flow hydrogenation of **11** over Pd@MonoBor catalyst (methanol solution 0.25 mL min^−1^, H_2_ 1.35 mL min^−1^ @ 2.2 bar, rt). Reprinted with permission from [136], © 2013 Elsevier.

An accurate study was carried out using the Pd@MonoBor monolithic catalyst [136], showing how the subtle effect of fine adjustments of concurrent flows of methanol substrate solution and H_2_ gas may tune the conversion and selectivity of the process. As anticipated for similar hydrogenation systems, under the same solution flow rate (i.e., keeping constant the residence time τ), an increase in the H_2_ flow rate (i.e., the H_2_/substrate molar ratio) resulted in a higher conversion and in a lower ene- and *Z*/*E* selectivity (Figure 10a). An increase in the solution flow rate (i.e., a decrease in τ) under a constant the H_2_/substrate molar ratio resulted in a conversion decrease, but in a selectivity enhancement (Figure 10b). A reproducible selectivity/conversion diagram could be drawn on this basis, as reported in Figure 10c. Best compromise results between selectivity and conversion resulted in 87% **11a** yield (95% ene selectivity, of which 93% *cis*, at 99% conversion) under mild conditions (294 K, residence time 42 s, ratio H_2_/**11** = 2.7, Table 2, entry 9). Pd@MonoBor is the first catalyst reported for the production of **11a** under continuous flow showing selectivity comparable to that of the industrial process, with additional benefits of lower noble metal content, no presence of toxic Pb or other additives [157]. The performance of Pd@MonoBor also compared favourably with that of other conventional batch systems [158–159]. Selectivity enhancement in the batch alkynes semi-hydrogenation was reported using egg-shell type catalyst. This was attributed to the short contact time of the intermediate alkene with the metal located on the catalyst surface, so that the alkene is quickly removed from the active phase with no possibility of further reduction [160]. A similar effect was invoked for Pd@MonoBor, whose site accessibility is hampered by the low swelling of the support in methanol solvent. Better swelling would allow the solvent to diffuse thoroughly the support, thereby producing a larger number of substrate–catalyst interactions, which results in lower alkene selectivity. The reaction using Pd@MonoBor was monitored for 14 h time-on-stream, showing no appreciable decay of conversion or selectivity (Figure 11). The catalyst could be reused several times with neither significant activity drop nor palladium leaching in solution detected.

Under comparable **11a** yield (76.9%), better productivity was shown by the packed Pd@titanate nanotubes catalyst (for **11a**: 40.6 mol g_Pd_^−1^ h^−1^, Table 2, entry 10) [137], analogously to what above described for the partial hydrogenation of **3**. As for Pd@MonoBor, the high selectivity observed in this case was attributed to the short contact time of the intermediate alkene with the Pd sites located onto the outer surface of the catalyst. Indeed, an excellent product yield was obtained for short residence times (13 s), that suggests a high contribution to catalytic activity by easy accessible PdNP. No catalyst efficiency decay was observed over 6 h time-on-stream.

**Figure 12:**
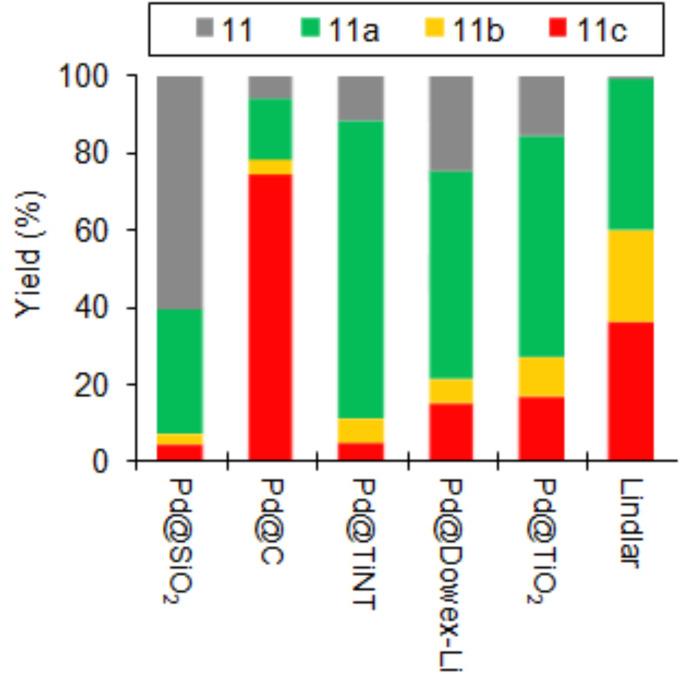
Continuous-flow hydrogenation reaction of **11** over packed-bed catalysts. Adapted with permission from [137], © 2016 John Wiley and Sons.

Worse performances were shown by other Pd packed-bed catalysts using conventional support materials, including mesoporous titania powder, mesoporous Davisil silica, Lindlar, gel-type Dowex resin, carbon (Table 2, entries 11–15), with the latter showing the highest rate of over-hydrogenation to 1-hexanol (**11c**, yield >70%, Figure 12) [136–137,161]. Good yield (68.2%) and productivity (11.4 mol g_Pd_^−1^ h^−1^) of **11a** were obtained by packing pellets of a hybrid zirconia/polyvinylalcohol matrix with embedded PdNP (Table 2, entry 16) [142].

**Figure 13:**
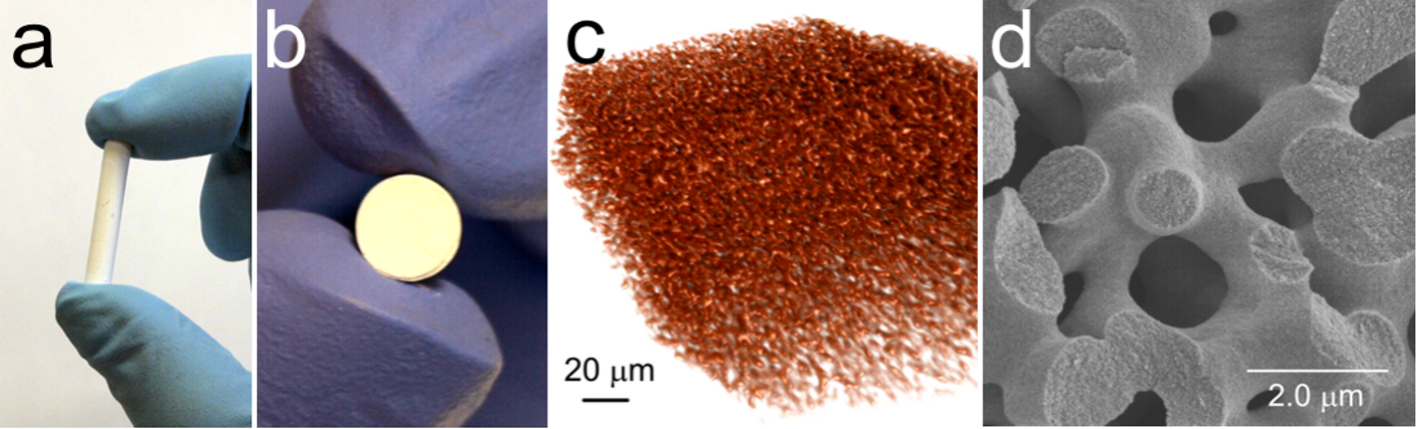
Images of the bimodal TiO_2_ monolith with well-defined macroporosity: (a, b) optical; (c) X-ray tomography; (d) scanning electron microscopy. Reprinted with permission from [163], © 2012 American Chemical Society.

Catalysts based on PdNP immobilized into the mesopores of hierarchically ordered meso- and macroporous inorganic silica [162] and titania monoliths [163] (Figure 13), were also reported showing moderate yields and remarkable catalyst stability over a period of 24 h (Table 2, entries 17, 18).

The heterogeneous hydrogenation of the parent alkyne substrate 3-phenyl-2-propyn-1-ol (**12**) is of interest because the corresponding alkene, the cinnamyl alcohol (**12a** + **12b**), is used in the formulation of perfumes and other personal care products [164]. The highest selectivity so far reported in batch conditions was observed using dendron-stabilized PdNP catalysts with quinoline additives (97% ene, 98% Z) [165]. The only example described under continuous flow used the Pd@monobor catalysts to achieve a 96% conversion and 79% selectivity to alkene and 95% to the *Z* isomer (Table 2, entry 19) [136].

#### 2-Butyne-1,4-diol

From an industrial point of view, there is a great interest in the selective semi-hydrogenation reaction of 2-butyne-1,4-diol (**13**) under flow conditions, since *cis*-2-butene-1,4-diol (**13a**) is an important intermediate in the synthesis of antibiotics, vitamins A and B_6_, several insecticides and antitumoral chemicals [166]. Currently, **13a** is manufactured from **13** in ca. 5000 t/y by a batch process under elevated pressures and/or temperatures, using 0.5% Pd@Al_2_O_3_ catalysts doped with Cd, Zn, Bi or Te [167]. On the laboratory scale, it is obtained with high selectivity (70–99% at 80–90% conversion) by the same route, using various supports and additives (including Zn, NH_3_, pyridine, KOH) [168–169].

Under the conditions of continuous-flow catalysis, Pd@MonoBor provided **13a** in moderate yields (70.2%) and high productivity (3.3 mol g_Pd_^−1^ h^−1^) at 294 K, with the formation of butyraldehyde byproducts in addition to the saturated alcohols **13c** and **13d**, as commonly reported in the literature for this substrate (Table 2, entry 20) [136].

Better product purity, but lower productivity, was observed using conventional flow reactors at higher reaction temperatures. Thus, Pd@Al_2_O_3_ catalysts, either as wash-coated honeycomb or as egg-shell particles packed-bed setups, resulted in a high ene- and *cis* selectivity (>93%) at 90% conversion at 328 K (Table 2, entries 21, 22) [170]. In that study, a comparison between honeycomb and packed-bed systems was carried out introducing the additional variables of cocurrent downflow contactor (CDC) and a trickle bed reactor (TBR) setups, that hinders a proper rationale of efficiency differences. However, the superior performance of honeycombs was highlighted both in terms of selectivity and productivity to **13a**. Supported Pd@Al_2_O_3_ was also reported by the group of A. N. Tsoligkas where a circular capillary reactor was used in co-current down-flow mode under Taylor flow regime (also known as slug and segmented flow) [171]. Selectivity to the *cis* isomer could be tuned by varying the liquid and the gas bubble slug length in that case. The optimized conditions showed slightly lower ene selectivity (91.4%) for this type of reactor. Similarly, commercial 5% Pd@charcoal operating in a slurry-type mode resulted in high selectivity in the presence of KOH and 323 K (Table 2, entry 23) [172].

As an alternative to palladium, a 1 wt % platinum catalyst supported onto calcium carbonate was also reported, however, with no practical advantages over Pd in terms of partial hydrogenation (27% ene selectivity at 78% conversion and 373 K) [173].

#### Methyl phenylpropiolate and 4-phenyl-3-butyn-2-one

The continuous hydrogenation of internal alkynes in presence of other functional groups other than alcohols was examined, for instance using the carbonyl derivatives methyl phenylpropiolate (**14**) and 4-phenyl-3-butyn-2-one (**15**). Both compounds were hydrogenated using the Pd@Monobor monolithic catalyst under mild conditions with >91% ene selectivity, and *cis* selectivity of 96% and 50%, respectively, at conversions higher than 90% (Table 2, entries 24, 25) [136]. Comparable selectivity results were obtained in batch using the Lindlar catalysts [174–175], Pd onto pumice [176] or onto nitrogen-doped carbon nanofibers [177], in the presence of 2.5–30% amine additives.

A variety of other single alkyne substrates have been hydrogenated under continuous-flow conditions using packed catalysts consisting of immobilized metal complexes. We refer the readers to the specific literature for these systems [178–179].

### Substrate scope

An explanation for efficiency differences observed in catalytic flow reactors in relation to the molecular structure and/or substituent groups of alkynes substrates is not apparent due to a number of reasons.

For example, although a higher selectivity in partial hydrogenation was reported for 1-hexyne (67%, **1**) compared to 1-heptyne (ca. 90%, **2**) under analogous conditions (30% conversion, room temperature, 1 bar H_2_, hydrocarbon solvent), hydrogenation experiments were carried out using different catalysts, namely 1% Pd@Al_2_O_3_ for **1** [121] and 2% Pd@Al_2_O_3_ for **2** [126], and reactor setups. Therefore, any effect of the alkyl chain length is to be considered with care in this case. Studies were reported for the continuous hydrogenation of 1-hexyne and 1-decyne by 16% CeO_2_@TiO_2_ catalyst under the same experimental conditions, showing a positive effect of chain length on selectivity (ca. 4% increase at full conversion) [119]. No justification for this evidence was proposed, however, a lower stability of the active site-adsorbed alkene intermediate with increasing steric hindrance may be hypothesized, which results in fewer interactions with hydrogen species, thus in enhanced the selectivity of the process [180].

**Figure 14:**
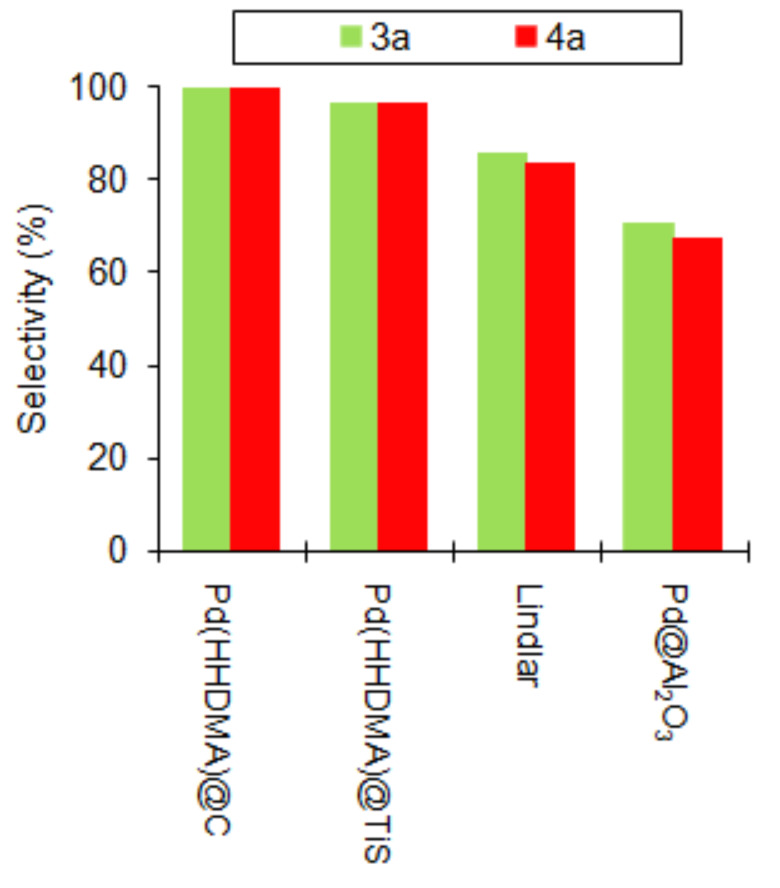
Selectivity of the continuous-flow partial hydrogenation reaction of **3** and **4** over packed-bed Pd catalysts at the same conversion level. Data from ref. [121].

On the other hand, a slightly negative effect of the alkyl substituent length on the selectivity of 2-methyl-3-butyn-2-ol (**3**) and 3-methyl-1-pentyn-3-ol (**4**) alcohols hydrogenation was demonstrated, for different Pd packed catalysts under the same conditions and substrate conversion [121]. Selectivity for **3** was equal or higher to that of bulkier **4**, irrespective whether Pd(HHDMA)@C, Pd(HHDMA)@TiS, Lindlar or Pd@Al_2_O_3_ catalyst was used (Figure 14).

Similarly, the hydrogenation of 2-methyl-3-butyn-2-ol (**3**) and 1,1-diphenyl-2-propyn-1-ol (**6**), bearing methyl and phenyl substituents, respectively, has been explored with a variety of catalytic flow reactors. While direct comparison in terms of substrate conversion is prevented by non-uniformity of reaction conditions, the dearth of a common trend emerges in terms of selectivity at the same level of conversion. An overall picture of experimental findings is summarized in Table 3. Selectivity for **6** is higher than that of **3** for Pd@TiNT and Lindlar packed catalysts, whereas the reverse is observed for monolithic Pd@MonoBor and packed Ag@SiO_2_. Given the large differences in the reactor systems, no rationale for these data can be hypothesized in the absence of theoretical or mechanistic studies.

**Table 3:** Relative selectivity in the continuous-flow partial hydrogenation reaction of **3** and **6** at comparable conversion level.

catalyst	alkyne		ref.

	**3**	**6**	

0.67% Pd@MonoBor^a^	higher		[136]
0.5% Pd@TiNT^b^		higher	[137]
5% Pd(Pb)@CaCO_3_^c^		higher	[123]
16% CeO_2_@TiO_2_^d^	equal		[123]
1.3% Ag@SiO_2_^e^	higher		[123]
1% Au@TiO_2_^e^	equal		[123]

^a^ca. 90% conversion, 293 K, ca. 1.5 bar H_2_. ^b^ca. 75% conversion, 293 K, ca. 2.7 bar H_2_. ^c^30% conversion, 293 K, 1 bar H_2_. ^d^30% conversion, 413 K, 40 bar H_2_. ^e^30% conversion, 373 K, 10 bar H_2_.

Phenylacetylene (**7**), 1-bromo-4-ethynyl benzene (**8**) and 1-ethynyl-4-nitrobenzene (**9**) were hydrogenated using a Pd@C catalyst with trimodal pore-size distribution [152]. The chemoselectivity to the corresponding alkene product showed to follow the order **7** >> **8** > **9**, under the same reaction conditions and comparable conversion. This result may be attributed to the increasing stabilization of the intermediate alkene due to the electron-withdrawing properties of the alkyne substituents (nitro > bromo >> unsubstituted).

Data were reported for the partial hydrogenation of 3-hexyne (**10**) and the parent alcohol 3-hexyn-1-ol (**11**) by mean of Lindlar catalysts (Table 2, entries 2 and 13) [119,136]. At comparable substrate conversion, the selectivity was significantly lower for the latter. Based on DFT calculations, a similar effect was justified for the hydrogenation of alkynols in terms of strong adsorption of alcohols on the Pd surface, that increases the contact time with catalyst, thereby resulting in lower selectivity [121].

The continuous hydrogenation of 3-phenyl-2-propyn-1-ol (**12**), methyl phenylpropiolate (**14**) and 4-phenyl-3-butyn-2-one (**15**), bearing -CH_2_OH, -CO_2_CH_3_ and -COCH_3_ substituents at the 1-position of phenylacetylene moiety, respectively, was investigated using the Pd@MonoBor catalyst [136]. Although a conversion and productivity trend cannot be highlighted due to the lack of experimental data under the same reaction conditions (concentration, H_2_:substrate molar ratio), the ene selectivity showed to decrease in the order **15** > **14** > **12** at comparable conversions (90–96%), that can be related to the stabilization of the alkene product by the deactivating keto groups, despite a contribution of adsorption energy of alcohol group cannot be ruled out.

## Conclusion

The selective, partial hydrogenation reaction of C≡C bonds is a process of high relevance in the current manufacturing technology of a variety of intermediates for the fine-chemical industry. The conventional batch processes employing Pd catalysts are often problematic because of selectivity issues, need of toxic additives, high metal loadings and limited catalyst resistance. Ever increasing environmental and economic constraints have boosted the development of innovative catalytic materials and processes with improved performance and lower environmental impact.

Great advancements have been achieved in the recent years in the design of continuous-flow systems for alkynes partial hydrogenation, showing efficiency that often surpass that of the industrial protocols. Two main elements of comparison can be highlighted.

**Batch versus flow setup.** While experimental comparison can be easily carried out, continuous-flow reactors are practically advantageous with respect to the corresponding batch systems in the instance that the same catalyst produces at least the same amount of desired product per mole of metal and unit time, under similar reaction conditions. Most examples illustrated above show that this is indeed the case. Selectivities comparable to that of batch systems have been achieved using flow reactors, with the unquestionable advantage that no additives are usually required. Higher activity of continuous-flow versus stirred-tank batch reactors was attributed to faster molecular flow to and from the active sites, as a consequence of both the larger surface area of the catalyst in contact with the reagents (convective mass transfer), and a more efficient permeation of fluids through the material (diffusive mass transfer), which facilitates a reaction transition from a diffusion-controlled to kinetic-limited regime [163,181]. The non-accumulation of co-products adsorbed on the catalyst surface may also significantly contribute to the minimization of active site inhibition under the conditions of continuous flow [27].

**Flow reactor design.** Performance differences among different continuous-flow reactor designs are difficult to rationalize due to the number of additional variables related to the catalyst involved, which include:

- the role played by the supported metal, e.g., type, loading, MNP size, shape and location [182–183]),

- the role played by the support material, e.g., acid/base properties [184–185], morphology, grain size [186], porosity [187], strong metal–support interactions, swelling propensity [188]). In propyne hydrogenation, for instance, catalyst resistance was shown to decrease with increasing acidity of the support [189].

In order to establish a proper comparison, the experimental conditions should also be reproduced with care. In alkynes partial hydrogenation, the higher the conversion, the lower is the selectivity. Therefore, selectivity of different systems shall be compared at the same conversion level, or better, selectivity/conversion diagrams shall be obtained by investigation of appropriate operating windows in relation to reagents flow rates, residence time, hydrogen/substrate ratio. Comparison between different systems requires a systematic study enucleating the contribution of each of the above factors, which is usually hampered by the lack or inhomogeneity of experimental data. In practice, to the best of our knowledge, no such analysis was carried out comparing the same catalyst under analogous flow reaction conditions in different reactor setups for liquid phase alkynes hydrogenation. Comparison between monolithic and packed (crushed material) arrangements was reported for Pd@TiO_2_ monolith catalyst and cyclohexene hydrogenation [163].

Despite no general conclusion can be drawn, structured monolithic reactors have usually shown superior performance with respect of packed-bed systems [162–163]. Due to their high permeability, monolithic materials allow for high substrate flow rates, weight hourly space velocities and low H_2_ back-pressures. As a consequence, the residence time of the intermediate alkene formed by hydrogenation is very short and it is continuously removed from the active sites with limited chance of further reduction. This results in an enhanced selectivity and productivity of semi-hydrogenation compound. When conventional (packed) mesoporous heterogeneous catalysts are used, the substrate undergoes a significant interaction with the metal sites inside the pores. The intermediate alkene is not swept away fast enough and it can react again before leaving the catalyst, thus resulting in a preferential formation of over-hydrogenation product at the same conversion level [190].

The activity of Pd-based catalysts is acknowledged to increase with decreasing particle size [191–192]. However, controversial statements can be found in the literature regarding the catalyst selectivity [193–194]. In the case of 2-methyl-3-butyn-2-ol (**3**) hydrogenation, the optimal catalyst in terms of desired partial hydrogenation productivity was established to be based on cubic PdNP of 3–5 nm length [195]. The combination of small metal particle size (high activity) and high flow rates (short contact time), as, e.g., in 1D materials or macroporous monoliths, seems, therefore, beneficial for continuous-flow partial hydrogenation of alkynes. In conclusion, the identification of the most effective and versatile catalytic system is difficult, since the choice is ruled by a variety factors to be evaluated, including selected performance indicators (product purity, productivity) and technical/economic parameters (cost and lifetime of the catalyst, reproducibility, reaction conditions, e.g., temperature, hydrogen pressure).
